# Pachychoroid pigment epitheliopathy: long-term structural changes and risk factors for disease evolution

**DOI:** 10.1038/s41433-026-04410-7

**Published:** 2026-03-26

**Authors:** Federica Lo Cascio, Gabriele Monici, Chiara Olivieri, Francesco Petrillo, Paola Marolo, Pasquale Viggiano, Francesco Boscia, Michele Reibaldi, Enrico Borrelli

**Affiliations:** 1 Department of Surgical Sciences, University of Turin, Turin, Italy; 2 Department of Ophthalmology, “City of Health and Science” Hospital, Turin, Italy; 3 Department of Translational Biomedicine Neuroscience, University of Bari Aldo Moro, Bari, Italy; 4 Department of Ophthalmology, Faculty of Medicine, Biruni University, İstanbul, Turkey

**Keywords:** Predictive markers, Retinal diseases

## Abstract

**Purpose:**

Pachychoroid pigment epitheliopathy (PPE) is an early, often asymptomatic stage of the pachychoroid disease spectrum that may progress to central serous chorioretinopathy (CSC), pachychoroid neovasculopathy, or polypoidal choroidal vasculopathy. This study evaluated longitudinal changes in retinal pigment epithelium (RPE) lesions in PPE and identified baseline predictors.

**Design:**

Retrospective cohort study.

**Participants:**

Forty-five eyes (45 patients) with PPE.

**Methods:**

All patients underwent multimodal imaging, including near-infrared reflectance, optical coherence tomography (OCT), blue fundus autofluorescence, and OCT angiography. RPE alteration areas were quantified by projecting OCT B-scan delineations onto near-infrared images. Central subfield thickness (CST) and central choroidal thickness (CCT) were measured, along with choroidal thickness at four quadrants 500 µm and 1000 µm from the foveal centre.

**Main outcome measures:**

Change in total RPE lesion area at 1-year follow-up and baseline predictors of lesion progression.

**Results:**

At 1 year, total RPE lesion area increased significantly (median 0.81 mm² vs. 0.71 mm² at baseline; *p* < 0.001), with a median percentage increase of 10.5%. The number of discrete lesions remained unchanged. CST and CCT showed no significant longitudinal changes. Preliminary 2-year data (*n* = 13) demonstrated similar trends. Multivariable regression identified greater baseline CCT as independently associated with increased RPE lesion progression, whereas smaller baseline RPE areas were associated with a greater relative increase.

**Conclusions:**

PPE demonstrates progressive RPE alterations over time despite stable retinal and choroidal thickness. Greater baseline choroidal thickness and limited initial RPE involvement predict faster lesion expansion, supporting PPE as an active and evolving condition requiring regular multimodal monitoring.

## Introduction

The pachychoroid spectrum disease (PSD) is a relatively recent entity in the field of chorioretinal pathology. It encompasses a number of conditions that were initially defined as pachychoroid pigment epitheliopathy (PPE), central serous chorioretinopathy (CSC), pachychoroid neovasculopathy (PNV), and polypoidal choroidal vasculopathy (PCV) [1, 2]. More recently, this spectrum has been expanded to also include peripapillary pachychoroid syndrome (PPS), pachyvitelliform maculopathy (PVM) and pachychoroid macular atrophy (PMA) [3–5].

Although the diagnostic criteria for pachychoroid disease have evolved over time – moving beyond choroidal thickness alone – current definitions emphasise both structural and functional alterations of the choroid. Historically, a subfoveal choroidal thickness greater than 300 µm was considered essential for diagnosis [1]; however, this cutoff is no longer mandatory. Updated criteria now include the presence of pachyvessels (i.e., dilated Haller’s layer vessels) [6, 7], choroidal vascular hyperpermeability on indocyanine green angiography (ICGA) [8], attenuation of the inner choroidal layers (choriocapillaris and/or Sattler’s layer) [7, 9, 10], a relative increase in choroidal thickness in affected compared with unaffected areas [11], and retinal pigment epithelium (RPE) alterations in the absence of typical features of age-related macular degeneration (AMD) [12].

The term pachychoroid pigment epitheliopathy (PPE) was first introduced by Warrow et al. in 2013 to describe macular retinal pigment epithelium (RPE) alterations with associated increased choroidal thickness and no history of subretinal fluid accumulation [13]. Clinically, PPE is characterised by several RPE alterations detectable with retinal imaging, such as RPE mottling, small serous pigment epithelial detachments (PEDs), pachydrusen, and areas of RPE hyperplasia. It is frequently asymptomatic and often detected incidentally during routine ophthalmic evaluation. Because of its subtle presentation and the limited awareness of this condition among clinicians, PPE is often overlooked or misdiagnosed as other disorders, including early/intermediate AMD, pattern dystrophy, and punctate inner choroidopathy [14]. Of note, PPE is frequently detected in the asymptomatic fellow eyes of patients with unilateral CSC, PNV, or PCV, highlighting its role as an early pachychoroid manifestation and a potential risk factor for progression to more advanced forms [15].

Multimodal imaging plays a critical role in the diagnosis of PPE. Fundus autofluorescence (FAF) typically reveals areas of mottled hypoautofluorescence, sometimes interspersed with focal hyperautofluorescence. On structural OCT, these regions correspond to irregularities of the RPE/Bruch’s membrane complex, with occasional small PEDs and localised thickening. Disruptions of the ellipsoid and interdigitation zones, as well as thinning of the outer nuclear layer, may also be observed, indicating early photoreceptor damage [16–18].

Although PPE has traditionally been viewed as a stable, non-progressive condition, increasing evidence indicates that it can evolve over time, potentially progressing to CSC or neovascular complications [19, 20]. However, longitudinal data are limited, and the natural history of the disease remains incompletely understood. To address this gap, the aim of this study was to investigate the longitudinal progression of RPE alterations in patients with PPE using structural OCT, evaluating changes in lesion extent over time and identifying demographic, clinical and anatomical factors associated with disease progression.

## Methods

### Study participants

This study was a retrospective longitudinal analysis. Subjects aged 18 years or older with PPE in at least one eye were randomly selected from the medical records of the Medical Retina Clinic at the “Città della Salute e della Scienza” Hospital, University of Turin, Turin, Italy, among patients evaluated between 2022 and 2025. The study protocol was approved by the Ethics Committee of the “Città della Salute e della Scienza di Torino”. The research adhered to the tenets of the Declaration of Helsinki and the Health Insurance Portability and Accountability Act.

Eligible patients were required to have a baseline assessment between September 2022 and June 2024 to ensure at least a 1-year follow-up. In the presence of the 2-year follow-up, also this was included in the analysis. Exclusion criteria were as follows: (i) a history of CSC or the presence of a “hyperautofluorescent track” on FAF imaging, indicative of previous subretinal fluid accumulation; (ii) prior treatment in the study eye with photodynamic therapy (PDT) or intravitreal anti-VEGF injections; and (iii) a history of, or any evidence for, choroidal neovascularisation (CNV) or other retinal or optic nerve disorders.

All enrolled patients underwent a comprehensive ophthalmic assessment, including multimodal imaging, at both baseline and follow-up assessments. The diagnosis of PPE was established by the treating physician (EB) based on multimodal imaging, in accordance with previously published criteria [21]. In particular, the structural OCT features considered for diagnosis included pachyvessels, inner choroidal thinning, RPE irregularities (including PEDs, RPE mottling, RPE hyperplasia and pachydrusen) in the absence of subretinal fluid. Infrared reflectance criteria comprised reduced fundus tessellation and hyperreflective drusenoid lesions. En-face OCT findings included dilated outer choroidal vessels corresponding to pachyvessels, as well as subtle RPE alterations.

The imaging modalities included in this study were obtained with the Spectralis HRA + OCT (Heidelberg Engineering, Heidelberg, Germany) device and include, as follows:

- Blue autofluorescence (BAF): BAF imaging was performed using a 488 nm excitation wavelength, with emission detected in the 500–700 nm range. The field of view was set to 30 × 30 degrees, with an image resolution of 768 × 768 pixels. Multiple single BAF frames were automatically aligned and averaged using the manufacturer’s software to enhance the signal-to-noise ratio.
- Spectral-domain optical coherence tomography (SD-OCT): structural SD-OCT scans were obtained using a volumetric scan protocol covering a 20 × 20-degree area, consisting of 49 horizontal B-scans with 16-frame averaging (ART Mean = 16), centred on the fovea. OCT scans were included in the analysis only if they met a minimum signal strength of 25, as recommended by the manufacturer [22]. In addition, a radial enhanced depth imaging (EDI) SD-OCT scan (6-mm, 6-line star pattern; ART mean = 16) centred on the fovea was acquired with a scan quality score ≥25 according to the manufacturer’s scale in order to be included in the study.
- Near-infrared reflectance (NIR): NIR imaging was acquired with the same device and field of view (30 × 30 degrees). It was performed simultaneously with SD-OCT to ensure spatial correspondence between modalities [23–25].

Swept-source optical coherence tomography angiography (SS-OCTA) was performed using the PLEX® Elite 9000 platform (Carl Zeiss Meditec Inc., Dublin, CA, USA) and was utilised to exclude cases with CNV [26–28].

### Grading protocol

At baseline, cases were first reviewed for eligibility by an experienced and certified grader (EB).

Successively, an expert grader (FL), masked to patients’ demographic and clinical information, identified all areas of RPE changes associated with PPE—defined as regions showing RPE mottling, small serous PEDs, pachydrusen, or RPE hyperplasia—on volumetric OCT scans. These areas were delineated based on the onset and termination of RPE alterations irrespective of changes in the photoreceptor layer. An area of normally appearing RPE from the same patient was used as a healthy control during the grading process. Any remaining ambiguous cases were individually evaluated by the expert and certified grader (EB), who corrected any potential errors.

The borders of PPE-associated RPE changes identified on structural OCT B-scans were annotated on the corresponding NIR images, with the device automatically providing the precise correspondence between points on the SD-OCT and the NIR images, as previously described [25]. Lesion area was calculated by connecting the margins of the segments of the RPE changes projected onto the corresponding NIR image. This process was performed using the device’s built-in calliper tools.

This process was performed based on a document containing the guidelines as previously reported, along with examples of PPE identified through SD-OCT. The grader reviewed and discussed the document with an experienced and certified grader (EB) to ensure full understanding of the grading criteria. The results were expressed in µm and mm². The grader selected the best image for the study from the two available images. Once the grading process was completed, the experienced and certified grader (EB) meticulously reviewed all cases to assess the reliability of the process and to correct any possible errors. However, intra- and/or inter-grader repeatability was not assessed.

Central subfield thickness (CST) was obtained directly from the device’s analysis software using the volumetric SD-OCT scans. Choroidal thickness was manually measured at the subfoveal location (central choroidal thickness, CCT) and in the four quadrants (superior, inferior, nasal, and temporal) at 500 µm and 1000 µm from the foveal centre. Choroidal thickness was defined as the vertical distance from the outer border of the retinal pigment epithelium to the choroid–scleral interface. All measurements were performed on radial OCT scans using the device’s built-in calliper tools.

### Statistical analysis

Qualitative variables are presented as number of patients and percentage. For quantitative variables, the Shapiro–Wilk test was used to assess normality. Variables with a normal distribution are reported as mean ± standard deviation (SD), whereas non-normally distributed variables are reported as median and interquartile range (IQR). Parametric and non-parametric tests for paired samples were employed to compare quantitative variables between baseline and follow-up visits. Additionally, multiple regression analysis was performed to evaluate whether demographic and clinical characteristics were associated with the percentage change in RPE alterations between baseline and the 1-year follow-up (dependent variable).

## Results

### Characteristics of patients included in the analysis

A total of 45 patients (45 eyes) were included in the analysis, with 45 patients completing a 1-year follow-up and 13 patients completing a 2-year follow-up. The mean age was 65.7 years (range, 35–88 years), and the cohort comprised 31 men (68.9%) and 14 women (31.1%). At baseline, the mean best-corrected visual acuity (BCVA) was 0.04 LogMAR (range, 0–0.30). At the 1-year follow-up, mean ± SD BCVA was 0.05 ± 0.08 LogMAR (*p* = 0.425), and at the 2-year follow-up, it was 0.07 ± 0.12 LogMAR (*p* = 0.219).

In the fellow eye, 13 patients (28.8%) had CSC including 6 (13.3%) with associated type 1 CNV and 1 (2.2%) with aneurysmal type 1 (AT1) CNV. Six patients (13.3%) had AT1 CNV without evidence of CSC, consistent with PCV, and 10 patients (22.2%) had PNV. Additional findings in the fellow eyes included PPE in 6 patients (13.3%), PPS in 1 patient (2.2%), pachychoroid macular atrophy (PMA) in 2 patients (4.4%), combined PMA and PPS in 1 patient (2.2%), cystoid macular degeneration in 1 patient (2.2%), and retinal vein occlusion (RVO) in 1 patient (2.2%). Three fellow eyes (6.7%) were classified as normal. The status of the fellow eye was not included in the data analysis and therefore did not influence the results of the study.

If both eyes met the inclusion criteria, only the right eye was included in the analysis.

### Longitudinal changes in anatomical parameters

At the 1-year follow-up, the total area of RPE changes showed a significant increase compared with baseline values (median = 0.81 mm²; IQR = 1.35 vs. 0.71 = mm²; IQR = 1.06; *p* < 0.001). In contrast, the number of distinct areas with RPE alterations remained stable, with a median of 2 (IQR 1.0) at baseline and 2 (IQR 1.1) at 1 year (*p* = 0.57).

**Fig. 1:**
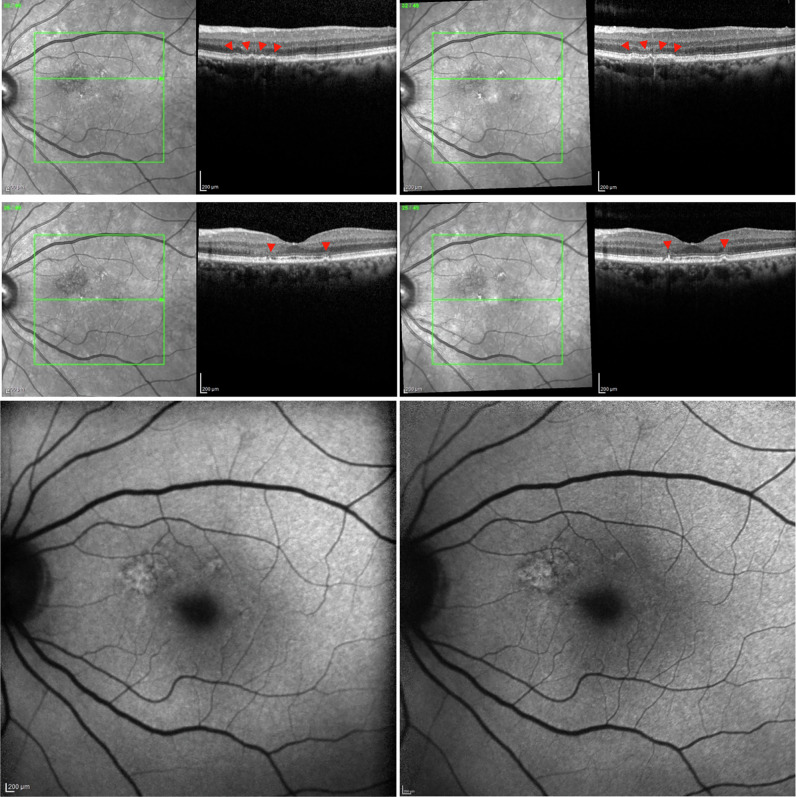
Clinical example of a patient with PPE showing enlargement of RPE alterations over a 1-year follow-up. Baseline (left column) and 1-year follow-up (right column) images from a patient with PPE. The NIR and structural OCT images (top and middle rows) reveal areas of RPE alteration (red arrowheads) that exhibit slight enlargement over time. Corresponding hyperautofluorescent lesions are visible on the BAF images (bottom row), further illustrating the progression of RPE changes.

The percentage change in the area size of RPE alterations between baseline and the 1-year follow-up showed a median increase of +10.5% (IQR = 18.6%) (Fig. 1).

Regarding thickness parameters, mean ± SD CST was 276.1 ± 18.1 µm at baseline and 273.6 ± 19.7 µm after 1 year, without a significant change (*p* = 0.12). Similarly, CCT was 350.6 ± 88.7 µm at baseline and 345.6 ± 90.6 µm at the 1-year follow-up visit (*p* = 0.09). Results of the topographical changes in choroidal thickness are presented in Table 1.

**Table 1:** Choroidal metrics observed in the study cohort across different visits.

w	Baseline	1-year follow-up	*P* value^a^	2-year follow-up	*P* value^b^
Central choroidal thickness, µm	350.6 ± 88.7	345.6 ± 90.6	0.088	370.7 ± 87.5	0.592
Temporal parafoveal choroidal thickness, µm	331.1 ± 94.3	328.4 ± 96.6	0.601	351.1 ± 81.7	0.764
Nasal parafoveal choroidal thickness, µm	325.8 ± 93.5	324.5 ± 96.4	0.814	370.1 ± 85.5	0.266
Superior parafoveal choroidal thickness, µm	329.0 ± 87.4	325.2 ± 91.2	0.316	365.0 ± 85.9	0.765
Inferior parafoveal choroidal thickness, µm	325.9 ± 99.3	321.2 ± 96.2	0.221	363.7 ± 92.1	0.578
Temporal perifoveal choroidal thickness, µm	312.4 ± 87.6	306.3 ± 96.2	0.108	339.9 ± 84.3	0.986
Nasal perifoveal choroidal thickness, µm	311.4 ± 89.5	298.7 ± 95.4	0.066	334.1 ± 81.8	0.544
Superior perifoveal choroidal thickness, µm	323.6 ± 83.2	317.7 ± 86.8	0.252	368.6 ± 77.6	0.775
Inferior perifoveal choroidal thickness, µm	314.3 ± 93.4	307.7 ± 91.3	0.110	355.7 ± 100.5	0.963

Quantitative values are expressed in mean ± SD (standard deviation).

^a^*p* value for baseline vs. 1-year follow-up comparisons.

^b^*p* value for baseline vs. 2-year follow-up comparisons.

As noted above, only 13 patients had data available at the 2-year follow-up. The percentage change in the area of RPE alterations between baseline and the 2-year follow-up showed a median increase of +110.0% (IQR = 28.6%). Given the limited sample size, the 2-year statistical analysis was performed on these 13 patients (13 eyes) and should be considered exploratory.

### Determinants of percentage change in RPE alterations

A multiple regression analysis was conducted to evaluate potential factors associated with the percentage change in the area of RPE alterations between baseline and the 1-year follow-up (dependent variable). Among the baseline parameters, greater CCT was independently associated with a larger increase in RPE area (SE = 1.533; *p* = 0.004) (Table 2). A smaller baseline RPE area was associated with a greater increase in RPE size over 1 year (SE = −1.320; *p* = 0.006), suggesting that patients with larger RPE areas at baseline tend to experience slower progression.

**Table 2:** Results of multiple regression analysis assessing the association between percentage change in RPE alterations and baseline variables.

	1-year percentage change in RPE alterations as dependent variable	
	Standardised estimate	*P* value
Age	0.016	0.921
Disease in the fellow eye	0.041	0.823
Total area of RPE changes	−1.320	0.006
Number of distinct areas with RPE alterations	0.037	0.837
Central subfoveal thickness	0.122	0.436
Central choroidal thickness	1.533	0.004
Temporal parafoveal choroidal thickness	−0.742	0.147
Nasal parafoveal choroidal thickness	−0.334	0.055
Superior parafoveal choroidal thickness	0.555	0.199
Inferior parafoveal choroidal thickness	-0.029	0.937

*RPE* retinal pigment epithelium.

## Discussion

In this longitudinal retrospective study, we assessed the progression of RPE alterations in eyes affected by PPE. In addition, we explored potential baseline biomarkers linked to the evolution of these changes to gain deeper insight into the natural history and determinants of disease progression. Our findings revealed a significant increase over time in the total area of RPE alterations, indicating that PPE—although frequently asymptomatic and apparently stable in the short term—can undergo structural progression. Moreover, we identified a positive association between baseline choroidal thickness and the subsequent enlargement of RPE lesions, suggesting that eyes with thicker choroids may develop RPE alterations more rapidly, with a consequent faster enlargement of these lesions.

As mentioned above, PPE is clinically characterised by RPE alterations, although it is often asymptomatic and associated with good visual acuity. Our results are consistent with these observations, as patients in our cohort had good visual acuity values that remained stable throughout the two-year follow-up period [15]. However, patients with PPE may experience additional visual disturbances such as metamorphopsia, underscoring the need for a more comprehensive characterisation of this condition. More importantly, PPE may progress over time and develop into CSC. This was demonstrated by Yagi et al. [20], who followed 148 eyes with PPE in patients with unilateral CSC and observed that 16.7% of them developed CSC over a 6-year follow-up period. These findings suggest that PPE represents a dynamic and evolving disease that warrants further investigation and understanding. Notably, increased foveal choroidal thickness was identified as a major risk factor for the progression from PPE to CSC in these patients.

The choroid plays a key role in the pathogenesis of pachychoroid disorders, including PPE [11, 18]. The presence of pachyvessels is considered a hallmark of this disease spectrum [13]. The term “pachyvessel” was introduced to describe these enlarged choroidal vessels, although the mechanisms underlying their formation were initially unclear. More recent evidence suggests that pachyvessels may arise from remodelling of Haller’s layer, driven by altered pressure gradients across choroidal watershed zones [29]. Choroidal morphological changes have been extensively characterised using structural OCT, which often reveals enlarged choroidal veins (i.e., pachyvessels) originating from Haller’s layer, relative preservation of Sattler’s layer, and thinning or attenuation of the overlying choriocapillaris [1]. Using structural OCT, Warrow and Freund were the first to describe dilated choroidal in eyes with PPE [13]. These choroidal alterations are typically more pronounced in regions corresponding to RPE changes [6]. Notably, en face OCT studies have confirmed the presence of dilated outer choroidal vessels associated with intraretinal foci in PPE, the latter serving as a surrogate biomarker for RPE alterations [7]. Collectively, these findings support a strong association between choroidal and RPE changes in PPE.

Therefore, given the pivotal role of the choroid in patients with PPE, we performed a topographical analysis of choroidal thickness in this cohort. Notably, our findings indicate that choroidal metrics remained largely stable throughout the observation period. This stability may reflect the relatively constant nature of the underlying choroidal vasculature in PPE. Similar findings have been reported in longitudinal studies of CSC and PNV, where choroidal parameters generally remain stable even after the resolution of subretinal fluid or following anti-VEGF therapy [30, 31].

In our study cohort, at the 1-year follow-up, the total area of RPE alterations significantly increased compared with baseline, despite the number of discrete lesions remaining stable. This indicates that disease progression in PPE is primarily driven by the gradual enlargement of pre-existing RPE changes rather than the formation of new ones. These findings support the notion that PPE may represent an early yet dynamic stage within the pachychoroid disease spectrum. Although traditionally regarded as a non-exudative and non-neovascular condition, our results suggest that RPE damage may progressively accumulate, potentially predisposing to more advanced manifestations of pachychoroid disease [20, 32, 33].

One of the most notable findings of our study was the association between baseline choroidal thickness and the subsequent enlargement of RPE alterations. Although increased choroidal thickness is no longer regarded as a diagnostic criterion, our multiple regression analysis revealed that thicker baseline choroids were positively correlated with the later expansion of RPE lesions. This suggests that greater choroidal thickness may predispose eyes to a faster enlargement of RPE alterations. These results are consistent with previous studies indicating that choroidal hyperpermeability and elevated hydrostatic pressure may contribute to RPE stress and dysfunction [34, 35].

Interestingly, smaller areas of RPE alterations exhibited a faster percentage increase in RPE changes over time. Several factors may account for this observation. First, this could reflect a mathematical effect: when the initial area is small, even a modest absolute increase results in a large percentage change. Second, smaller lesions have a higher perimeter-to-area ratio, leading to proportionally greater growth relative to their total size. Finally, smaller lesions may represent earlier and more active stages of the disease, whereas larger lesions could correspond to more mature or stabilised forms that progress more slowly.

In our study cohort, none of the patients developed additional manifestations of the pachychoroid disease spectrum beyond PPE during the observation period, although, as noted above, progression to other PSD disorders (e.g., CSC) is not uncommon in patients with PPE [20]. This absence of progression may be partly explained by the relatively short follow-up, which might have been insufficient to capture the development of other pachychoroid entities. Moreover, although PPE shares several pathophysiologic features with other forms of PSD, subtle differences in underlying mechanisms may exist [2, 11]. As a result, eyes with PPE might not necessarily evolve toward other manifestations. These distinctions likely extend beyond mere variations in choroidal thickness, warranting further longitudinal and mechanistic studies to clarify the specific determinants of disease progression.

Our study has several limitations that should be acknowledged. First, the small sample size—particularly at the 2-year follow-up—and the relatively short observation period may have limited our ability to detect progression to other pachychoroid disease entities. Additionally, the only functional parameter assessed was BCVA, which primarily reflects foveal function and may not capture more subtle visual changes that could be relevant when interpreting longitudinal RPE modifications. In future studies other functional parameters (e.g., metamorphopsia, localised sensitivity impairment) associated with RPE alterations may be evaluated in order to assess functional changes in PPE. In our cohort, most fellow eyes exhibited more advanced pachychoroid conditions, such as CSC or PNV, while only a minority showed PPE or no abnormalities. This distribution likely reflects a selection bias toward patients presenting with clinically significant disease in the fellow eye. Consequently, asymptomatic individuals with bilateral PPE, or with PPE in one eye and a normal fellow eye, are underrepresented. Therefore, we cannot exclude the possibility that cases with severe fellow-eye pachychoroid disease progress more rapidly, although our multivariate analysis did not detect such an association; however, the study may have been underpowered to adequately assess this.

In conclusion, our findings indicate that PPE is a dynamic condition characterised by progressive structural changes over time, particularly in eyes with greater baseline choroidal thickness. Larger and longer-term prospective studies incorporating detailed macular function assessments are needed to elucidate how these longitudinal changes influence visual function and whether they may predispose to progression from PPE to other entities within the pachychoroid disease spectrum.

## Summary

### What was known before

- Pachychoroid pigment epitheliopathy (PPE) is considered an early, often asymptomatic stage of the pachychoroid disease spectrum but may progress to CSC, PNV, or PCV.
- RPE alterations in PPE are recognised, but their long-term behaviour and factors predicting progression have been poorly characterised.

### What this study adds

- PPE shows measurable, progressive enlargement of RPE lesions over time, and greater baseline choroidal thickness as well as smaller initial RPE involvement predict faster lesion expansion.

## Data Availability

Data are available upon reasonable request from the corresponding author.
